# Sexually Dimorphic Immune and Neuroimmune Changes Following Peripheral Nerve Injury in Mice: Novel Insights for Gender Medicine

**DOI:** 10.3390/ijms22094397

**Published:** 2021-04-22

**Authors:** Valentina Vacca, Sara Marinelli, Federica De Angelis, Daniela F. Angelini, Eleonora Piras, Luca Battistini, Flaminia Pavone, Roberto Coccurello

**Affiliations:** 1 CNR-National Research Council, CNR, Institute of Biochemistry and Cell Biology, Monterotondo Scalo, 00015 Rome, Italy; lale_84@libero.it (V.V.); sara.marinelli@cnr.it (S.M.); fede75deangelis@gmail.com (F.D.A.); 2 IRCCS Fondazione Santa Lucia, 00143 Rome, Italy; df.angelini@hsantalucia.it (D.F.A.); elepiras23@gmail.com (E.P.); l.battistini@hsantalucia.it (L.B.); 3 CNR-National Research Council, CNR, Institute for Complex System (ISC), via dei Taurini 19, 00185 Rome, Italy

**Keywords:** neuropathy, microglia, astrocytes, T-cells, chemokines, cytokines, leptin, neuropathic pain, estrogen

## Abstract

Neuropathic pain (NeP) in humans is often a life-long condition with no effective therapy available. The higher incidence of female gender in NeP onset is worldwide reported, and although the cause is generally attributed to sex hormones, the actual mechanisms and the players involved are still unclear. Glial and immune cells take part in NeP development, and orchestrate the neuroimmune and inflammatory response, releasing pro-inflammatory factors with chemoattractant properties that activate resident immune cells and recruit immune cells from circulation. The neuro-immune crosstalk is a key contributor to pain hypersensitivity following peripheral nervous system injury. Our previous works showed that in spite of the fact that female mice had an earlier analgesic response than males following nerve lesion, the recovery from NeP was never complete, suggesting that this difference could occur in the very early stages after injury. To further investigate gender differences in immune and neuroimmune responses to NeP, we studied the main immune cells and mediators elicited both in plasma and sciatic nerves by peripheral nerve lesion. After injury, we found a different pattern of distribution of immune cell populations showing either a higher infiltration of T cells in nerves from females or a higher infiltration of macrophages in nerves from males. Moreover, in comparison to male mice, the levels of cytokines and chemokines were differently up- and down-regulated in blood and nerve lysates from female mice. Our study provides some novel insights for the understanding of gender-associated differences in the generation and perseveration of NeP as well as for the isolation of specific neurodegenerative mechanisms underlying NeP. The identification of gender-associated inflammatory profiles in neuropathy is of key importance for the development of differential biomarkers and gender-specific personalized medicine.

## 1. Introduction

Neuropathic pain (NeP) is a major problem in clinical practice, considerably affecting patients’ quality of life. Despite extensive research, the pathophysiological mechanisms underlying NeP are still not fully understood and no effective pharmacological treatments are available.

Increasing evidence indicates that glial and immune cells regulate nociceptive signaling in different anatomical regions, including the peripheral and central nervous systems [1,2,3,4,5]. Inflammatory mediators (pro-inflammatory cytokines and chemokines) released from immune cells can stimulate pain-relevant sensory afferent fibers either by a direct action or indirectly by stimulating other inflammatory mediators. Anti-inflammatory cytokines, which are also released by immune and immune-like cells, may play a role in reducing pain and in the homeostasis of the system [3,6,7].

Numerous clinical [8,9,10,11,12] and preclinical [13,14,15,16,17,18,19] studies have increasingly recognized how sex differences play a fundamental role in pain. Moreover, sex-related immune cells activation in pain processing is continuously emerging, with the immune system playing a major role in expression, progression and maintenance of chronic pain [6,7,20,21,22]. Differences between males and females in immune system cell populations emerged in chronic pain from inflammation and nerve injury, strongly suggesting the importance of considering both sexes in the investigation of modulatory role of immune system in pain [6]. Sorge and co-workers [23] showed that microglia exert a critical role in chronic pain processing in male mice, while the same role appeared to be played by T cells in females. On this subject, it is interesting to note that we observed [16] in the Chronic Constriction Injury (CCI) model of NeP an activation of microglia also in female mice, but with a different time-interval from nerve ligation than in males. Indeed, male mice showed gliosis after few days from ligation, while females showed a late activation of glial cells that persisted for a long time.

The modulation of gonadal hormones by the immune response highly influences sexual dimorphism, with estrogens being generally considered immunostimulatory and testosterone immunosuppressive [22,24]. Considering this point, we extended our study to neuropathic female mice by investigating the effects of 17β-Estradiol treatment on behavioral and immune responses. The results of the present research may lead to a better understanding of the mechanisms underlying sex-related differences in chronic pain and help to disclose some points for the comprehension of the interaction between sex, immune system and NeP. The possibility to identify sex-associated immune biomarkers is of great relevance for the development of novel diagnostic tools and personalized therapies in pain disorders, in which the current approaches are largely unsatisfactory.

## 2. Results

### 2.1. Sex Differences in Neuropathic Pain Development and Involvement of Immune System

#### 2.1.1. Functional Testing: Sex-Differences in Mechanical Nociceptive Threshold

**Figure 1:**
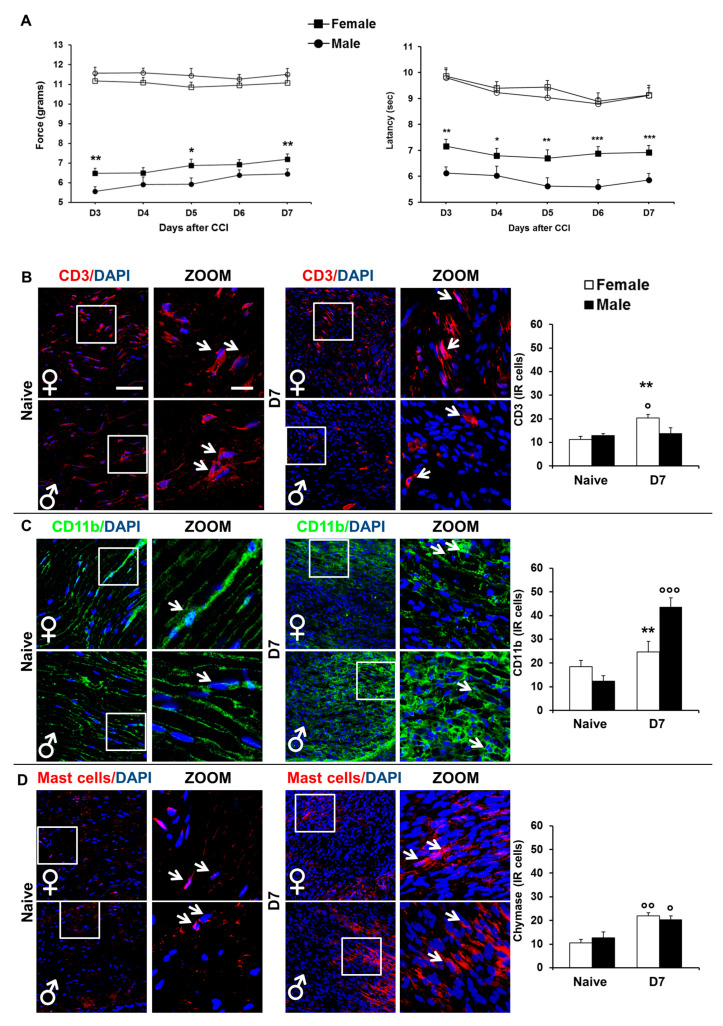
(**A**) left panel. Mechanical nociceptive threshold to neuropathic pain in female and male mice. Sex-related differences in the development of mechanical allodynia induced by CCI: time course of withdrawal thresholds (expressed as applied force in grams) of hind paws ipsi- and contralateral to the injury in female (ipsi ■, contra □) and male (ipsi ●, contra ○) mice. Numbers of mice for the two different experimental groups were: *n* = 20 (male), and *n* = 20 (female). (*) *p* < 0.05, (**) *p* < 0.01 between male and female mice. (**A**) right panel. Thermal hyperalgesia threshold to neuropathic pain in female and male mice. Time course of thermal hyperalgesia (expressed as latency in seconds) of hind paws ipsi- and contralateral to the injury in female (ipsi ■, contra □) and male (ipsi ●, contra ○) mice. Numbers of mice for the two different experimental groups were: *n* = 20 (male), and *n* = 20 (female). (*) *p* < 0.05, (**) *p* < 0.01, (***) *p* < 0.001 between male and female mice. (**B**–**D**) Sex differences in the expression of several immune positive cells in sciatic nerve. Representative examples of high magnification (63×) IF images of several immune positive cells and nuclei (DAPI, blue), taken from sciatic nerves of no CCI (naïve, left panels) and CCI D7 (D7, right panels) male and female mice, respectively. Square indicates the area considered for acquisition of the representative examples of high magnification (63× with 3× zoom) IF images reported in other panels (scale bar: 50 μm). (**B**) CD3 (limphocytes T positive cells, red). Histograms show the quantification of the total number of immune positive cells in male and female no CCI and CCI (D7) mice. (**) *p* < 0.01, between male and female mice; (°) *p* < 0.05, vs. the corresponding naïve group. (**C**) CD11b (macrophages positive cells, green). Histograms show the quantification of the total number of CD11b positive cells in male and female no CCI and CCI (D7) mice. (**) *p* < 0.01, between male and female mice; (°°°) *p* <0.001, vs. the corresponding naïve group. (**D**) Mast cell chymase (Mast positive cells, red). Histograms show the quantification of the total number of mast cell chymase positive cells in male and female no CCI and CCI (D7) mice. Histograms show the quantification of the total number of CD11b positive cells in male and female no CCI and CCI (D7) mice. (°) *p* < 0.05, (°°) *p* < 0.01, vs. the corresponding naïve group.

We previously demonstrated [16] that in spite of the initial better response after NeP induction, female mice never exhibited a complete functional recovery as compared to males. Here, we performed again the experiment considering the first 7 days from the peripheral nerve damage, the period in which the local inflammatory response is higher. To assess sex-differences in NeP development we subjected female and male CD1 mice to CCI. Withdrawal thresholds to mechanical stimuli, induced by Aesthesiometer plantar test, were observed in experimental groups. Figure 1A shows that, after CCI, the mechanical nociceptive threshold decreases, in both male and female mice, by 40–50% in the ipsilateral, as compared to the contralateral hind paw. However, the time course of allodynic response was different between females and males, with neuropathic female mice showing a higher withdrawal threshold of the injured paw than male neuropathic mice.

Two-way ANOVA for repeated measures revealed significant main effects for sex (F1,38 = 10,766; *p* = 0.0022) and time (F4,152 = 8.532; *p* < 0.0001), but not for sex-time interaction (F4,152 = 0.728; *p* = 0.5743). Two-way ANOVA for repeated measures didn’t show significant differences in withdrawal of contralateral paws.

In addition, we evaluated the development of thermal hyperalgesia (Figure 1A, right panel) and tested the experimental groups as for their response to plantar test (acute nociceptive thermal threshold), confirming a similar trend of allodynia in hyperalgesia response. Significant differences were observed for both sexes in comparison with contralateral hindpaw and between male and female mice in the ipsilateral threshold for the entire period analyzed.

Two-way ANOVA for repeated measures revealed significant main effects for sex (F1,38 = 15.241; *p* = 0.0004) and time (F4,152 = 6.268; *p* = 0.0001), but not for sex-time interaction (F4,152 = 1.473; *p* = 0.213). Two-way ANOVA for repeated measures did not show significant differences in the withdrawal of contralateral paws.

#### 2.1.2. Immunohistochemical Analysis: Sex-Differences in the Expression of Immune Positive Cells in Sciatic Nerve

By means of confocal microscopy analysis of immunofluorescent markers, we evaluated the expression and morphology of different immune cells generally expressed in the sciatic nerve in the first days of Wallerian degeneration (WD). As expected, before CCI, no significant sex-related differences for the immune cells analyzed were observed (Figure 1B). By contrast, after injury, in CCI D7 female mice there was a significant infiltration in the sciatic nerve of T cells (CD3) that was more expressed than in both naïve females and CCI D7 male mice (*p* = 0.007 and *p* = 0.0105, respectively). On the other hand, after injury we observed a higher infiltration of macrophages in CCI D7 male mice than in naïve non-CCI males (*p* = 0.0055), while females did not differ in the expression of Cd11b before and after injury. These data were confirmed by flow cytometry analysis carried out after injury, which showed a major infiltration of T cells and macrophages in females and males, respectively, as compared to naïve conditions (see Section 2.1.3). Compared to naïve condition, a major infiltration of mast cells (i.e., revealed by chymase expression) was evident after injury in both sexes, which however did not differ from one another.

#### 2.1.3. Flow Cytometry Analysis for Immune Markers on Sciatic Nerve Lysate and Plasma Levels

**Figure 2:**
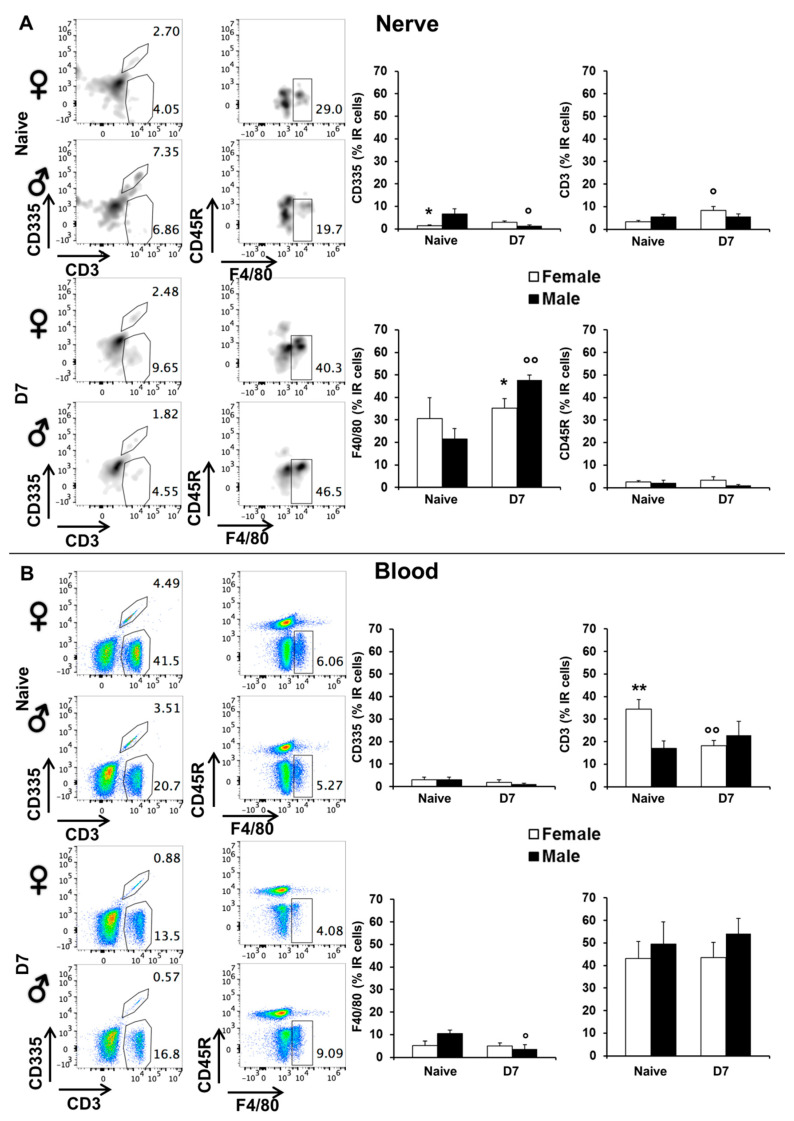
Flow cytometry. Panels show representative examples FACs plots. The following anti mouse antibodies were used: CD45 APC-eFluor-780 conjugated to select hematopoietic lineage cells; CD335 PE-eFluor 610 conjugated characteristic of Natural Killer cells; CD3 PE conjugated to identified T lymphocytes; CD45R VioBlue conjugated to identified B lymphocytes; F4/80 PE Vio770 conjugated and MHC-II Fitc conjugated to select monocyte population. Histograms show the percentages of several immune cells present in serum and sciatic nerve of male (dashed black bars) and female (dashed white bars) mice at both time points: naïve and D7 post CCI. (**A**) Panels show the percentages of several immune cells present in serum and sciatic nerve of male and female mice in naïve condition. Histograms show the percentages of several immune cells present in serum. (*) *p* < 0.05 between male and female mice; (°) *p* < 0.05; (°°) *p* < 0.01, vs. the corresponding naïve group. (**B**) Panels show the percentages of several immune cells present in blood and sciatic nerve of male and female seven days post CCI. Histograms show the percentages of several immune cells present in sciatic nerve. (**) *p* between male and female mice; (°) *p* < 0.05, (°°) *p* < 0.01, vs. the corresponding naïve group.

To determine and quantify immune cells present in sciatic nerve and in the blood flow, the Flow cytometry was performed before (naïve) and seven days after CCI (D7) on sciatic nerve-derived cells (Figure 2A), and from plasma (Figure 2B) stained with antibodies against CD335 (Natural killer positive cells), CD3 (T positive cells), F4/80 (macrophages positive cells) and CD45R (B positive cells). Depending on the cell population considered, we found before and/or after injury sex-associated differences. FACs plots and histograms revealed a significantly lower presence of Natural Killer (NK) positive cells in naïve female than in naïve male mice (*p* = 0.0156). After injury, a significant decrease of NK positive cells was observed in male mice (*p* = 0.0138 vs. naïve male mice), thus becoming similar to those observed in females (Figure 2A). On the other hand, at D7 there was a significant increase of infiltrated T cells in the sciatic nerve of female mice as compared to naïve females (*p* = 0.0109); no changes were detectable in male groups. We also observed a greater infiltration of macrophages in CCI D7 males than CCI D7 females (*p* = 0.031) and naïve male mice (*p* = 0.0221, Figure 2A). We did not observe any sex-related differences after injury for lymphocytes B.

FACS plots on plasma specimens (Figure 2B) show sex-associated differences in CD3 positive cells (lymphocytes T), which were higher in females than in males in naïve condition (*p* = 0.0123) and significantly reduced after injury (*p* = 0.0128). The presence of macrophages in male mice is significantly reduced after injury, thus differing from what observed in the sciatic nerve where an increase of the F4/80 marker was detected. The presence of NK and B positive cells was not altered after CCI and did not show sex-related differences.

#### 2.1.4. Inflammatory Modulators and Sex Differences

It was previously shown that seven days after CCI in male mice there is a different up- and down- regulation of inflammatory mediators, in both serum and sciatic nerve lysates compared to naïve condition [25]. To study sex-related differences in the cytokines/chemokines levels, we used the same panel of 40 pro- and anti-inflammatory mediators in sciatic nerve lysates and serum of female mice at two different time points, before (naïve) and seven days post CCI (D7). In sciatic nerve lysates of CCI females (Table 1), we found an up-regulation of several pro-inflammatory agents, such as IL1β, IL12p40/70, I-TAC (Interferon inducible T cell alpha chemoattractant, also known as CXCL11), KC, MIP1-α and leptin (an adipokine appetite-regulatory hormone).

The anti-inflammatory cytokine IL-10 and IL-13, which plays a role in inflammatory and autoimmune disorders as well as in NeP, was also upregulated.

Moreover, we observed an up-regulation of cytokines/chemokines involved in immune activation/recruitment, such as: (i) CD30L, a mast cells activator; (ii) GCSF chemokines, important for differentiation of granulocytes and Fas ligand, which play a considerable role in the regulation of the immune system.

The table shows only significant inflammatory mediators revealed in sciatic nerve samples of Naïve or CCI (D7) female mice. Data are shown as FOLD CHANGE (CCI/NAÏVE). Any >1. 5-fold increase or <0. 65-fold decrease in signal intensity for a single analyte between samples may be considered a measurable and significant difference in expression. Values indicate the significant upregulation and downregulation versus naïve.

On the other hand, we observed a down-regulation of some anti-inflammatory cytokines/chemokines expression, as follows: (i) BCL (also known as CXCL13) that promotes the migration of B lymphocytes; (ii) MIG (Monokine induced by Interferon Gamma, also known as CXCL9) that influences the initial recruitment of macrophage/microglia; (iii) fractalkine (also known as CX3CL1) that after peripheral injury results substantially upregulated in spinal microglia; (iv) SDF-1 (stromal cell derived factor 1, also called CXCL12) that plays essential roles in the biological response (as activation/proliferation of glial cells) to peripheral nerve injury and in neuromodulation in the central nervous system. These chemokines affect peripheral sensitization through neuronal and glial mechanisms. At the same time, we observed a down-regulation of some pro-inflammatory cytokines/chemokines expression, as follows: (i) exotoxin-2, (ii) MIG and (iii) GM-CSF (granulocyte macrophage colony stimulating factor). Finally, there was a decrease of TNFα soluble receptors (Type I and II), which are endogenous inhibitors of TNFα (Table 1). When pro-inflammatory cytokines/chemokines were investigated in serum from females we observed an up-regulation of several molecules previously found down-regulated in male mice [25], such as IL1α, IL12p40/70, IL6, KC and MIP1-α, a chemokine that was also found higher in the blood of neuropathic pain patients [26] (Table 2). Other pro-inflammatory cytokines were also up-regulated, in particular: RANTES (also known as CCL5), I-TAC, MIG, SDF1, MCP1 (also known as CCL2), one of the key chemokines that regulate migration and infiltration of monocytes/macrophages, TIMP-2 (Metalloproteinase inhibitor 2) and IL-10. Fas ligand, which plays an important role in cell death regulation, and leptin were down-regulated in female serum samples (Table 2).

The table shows only significant inflammatory mediators revealed in sciatic nerve samples of Naïve or CCI (D7) female mice. Data are shown as FOLD CHANGE (CCI/NAÏVE). Any >1. 5-fold increase or <0. 65-fold decrease in signal intensity for a single analyte between samples may be considered a measurable and significant difference in expression. Values indicate significant upregulation and downregulation versus naïve.

#### 2.1.5. Immunohistochemical Analysis: Sex Differences in the Expression/Activation of Spinal Glial Cells

We performed IF staining of astrocytes (GFAP) and microglia (CD11b) before (naïve) and 7 days after CCI (D7) to analyze sex-related differences in the expression of glial cells, examining their activation and morphological features.

**Figure 3:**
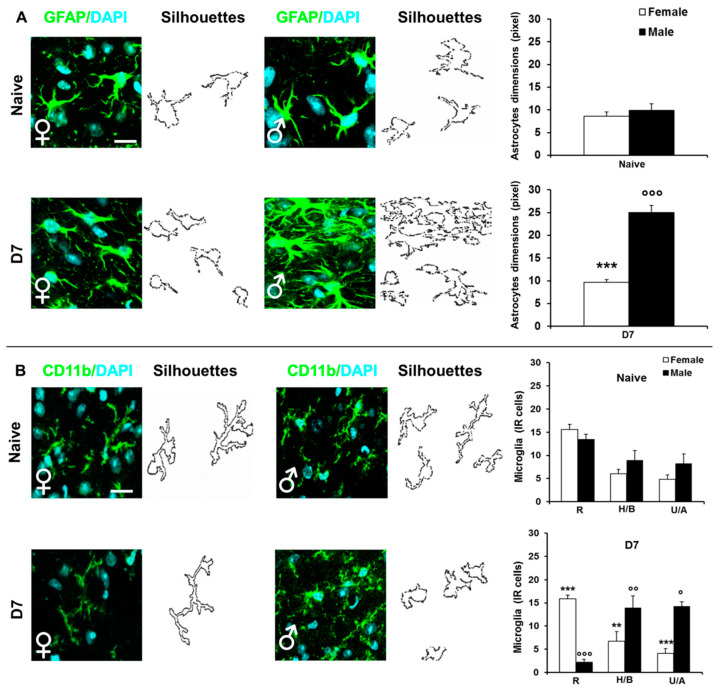
Sex differences in the expression/activation of spinal glial cells. Left panels show representative examples of high magnification (63× with 3× zoom) IF images showing the expression of: (**A**) GFAP (astrocytes) in L4/L5 spinal cord sections taken from no CCI (naïve) and CCI D7 (D7) male and female mice (scale bar: 50 μm). Each image has been transformed to a digital image where outline of cell silhouettes was identified and automatically measured for astrocytes by using RGB method that converted pixel in brightness values. Histograms show the quantification of astrocytes dimension in male (dashed black bars) and female (dashed white bars) and no CCI (naïve) and CCI (D7) mice. (***) *p* < 0.001 between male and female; (°°°) *p* < 0.001 vs. the corresponding naïve group. (**B**) CD11b (microglia) in L4/L5 spinal cord sections taken from no CCI (naïve) and CCI D7 (D7) male and female mice (scale bar: 50 μm). Each image has been transformed in digital image where outline of cell silhouettes was identified and singularly counted and divided about the different morphology: (Resting/Ramified (R), Hyperactive/Bushy (H/B), Unramified/Ameboid (U/A). Histograms show the quantification of the different morphology of CD11b positive cells in male (dashed black bars) and female (dashed white bars) no CCI (naïve) and CCI (D7) mice. (**) *p* < 0.01, (***) *p* < 0.001, between male and female; (°) *p* < 0.05, (°°) *p* < 0.01, (°°°) *p* < 0.001 vs. the corresponding naïve group.

In baseline condition, the size of astrocytes did not differ between female and male mice (Figure 3A) and we didn’t observe any difference in microglia of naïve animals, independently from the morphological phenotypes (R), (H/B), (U/A) (Figure 3B). Seven days after CCI, a significant increase of reactive and hyperactive astrocytes was present in male mice with respect to females (*p* < 0.001; Figure 3A). In addition, while CCI males showed a significant increase of (H/B) and (U/A) and a reduction of (R) microglia in comparison with naïve condition (*p* = 0.0351 H/B; *p* = 0.0429 U/A; *p* < 0.0001 R), female group had a similar IR cell number before and after CCI, causing each phenotype to significantly differ from the corresponding male group (H/B: *p* = 0.00026, U/A: *p* < 0.0001, R: *p* < 0.0001) (Figure 3B). These data confirm that neuropathy triggers a sex-dependent evolution of glia activation 16.

### 2.2. 17β-Estradiol Counteracts Neuropathic Pain in Female Mice

#### 2.2.1. Functional Testing: Mechanical Nociceptive Threshold

**Figure 4:**
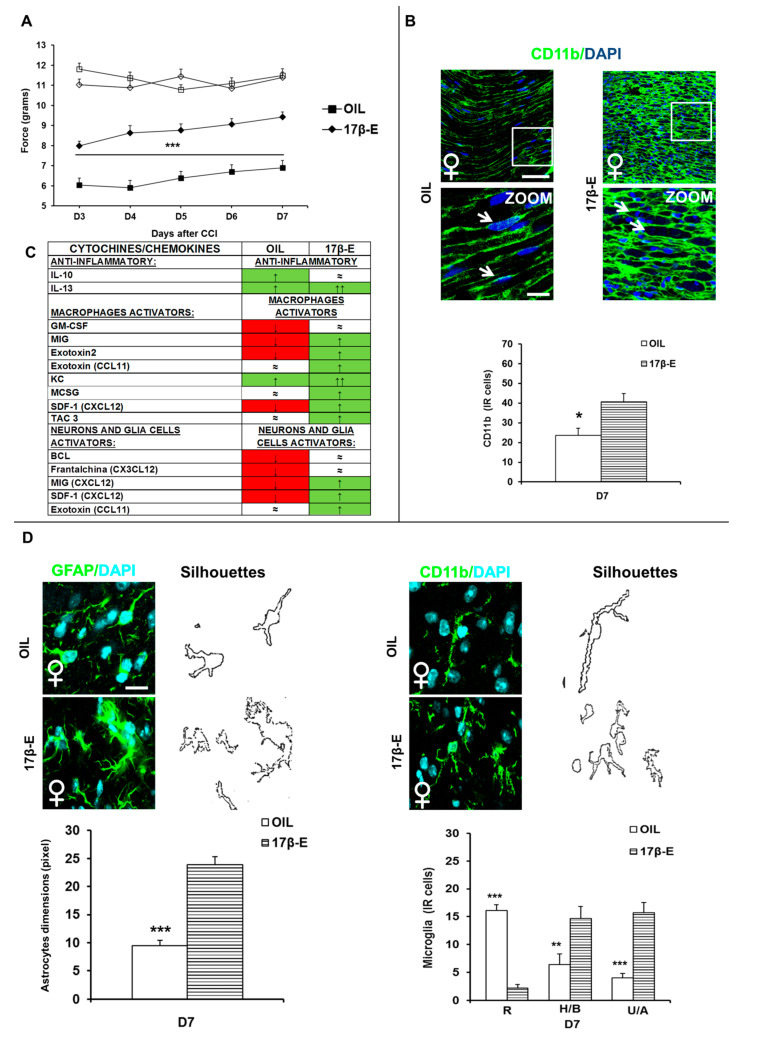
17β-estradiol neuroimmune modulation. (**A**) Mechanical nociceptive threshold to neuropathic pain in in control (OIL) and 17β-estradiol-treated female mice (17β-E). Time course of withdrawal thresholds (expressed as applied force in grams) of hind paws ipsi- and contralateral to the injury in OIL-female (ipsi ■, contra □) and 17β-female (ipsi ♦, contra ◊) mice. Numbers of mice for the two different experimental groups were: *n* = 10 (OIL), and *n* = 10 (17β-E). (***) *p* < 0.001 between OIL and 17β-E female mice. (**B**) Differences in the expression of macrophages positive cells in sciatic nerve. Representative examples of high magnification (63× and 63× with 3× zoom) IF images of CD11b (macrophages positive cells, green) and nuclei (DAPI, blue), taken from sciatic nerves of CCI D7 (D7) OIL and 17β-E female mice, respectively. Square indicates the area considered for acquisition of the representative examples of high magnification (63× with 3× zoom) IF images reported in other panels (scale bar: 50 μm). Histograms show the quantification of the total number of CD11b positive cells in OIL (dashed black bars) and 17β-E female (dashed white bars). (*) *p* < 0.05, between OIL and 17β-E female mice. (**C**) Cytokines’ modulation in nerves tissue lysates sample. The table shows a summary of most important up (↑, green), down-regulated (↓, red) and equal (≈, white) cytokines. The cytokines up-regulate. (**D**) Expression/activation of spinal glial cells. Left panels show representative examples of high magnification (63× with 3× zoom) IF images showing the expression of: GFAP (astrocytes) and CD11b (microglia) in L4/L5 spinal cord sections taken from CCI D7 (D7) OIL and 17β-E female mice (scale bar: 50 μm). Each image has been transformed in digital image where outline of cell silhouettes was identified and automatically measured for astrocytes by using an RGB method that converted pixel in brightness values. Histograms show the quantification of astrocytes dimension in OIL (white) and 17β-E female (dashed white bars) CCI (D7) mice. (***) *p* < 0.001, between OIL and 17β-E female mice. For microglia each image has been transformed in digital image where outline of cell silhouettes was identified and singularly counted and divided about the different morphology: (Resting/Ramified (R), Hyperactive/Bushy (H/B), Unramified/Ameboid (U/A). Histograms show the quantification of the different morphology of CD11b positive cells in OIL (dashed black bars) and 17β-E female (dashed white bars) CCI (D7) mice. (**) *p* < 0.01, (***) *p* < 0.001, between OIL and 17β-E female mice.

Figure 4A shows the mechanical nociceptive thresholds in control (OIL) and 17β-estradiol-treated female mice (17β-E) after CCI. Although sciatic nerve ligation induced a reduction of mechanical threshold in both groups, a different threshold and time course was nevertheless evident. The treatment with 17β-estradiol partially counteracted the effects of CCI, enhancing the withdrawal threshold of the injured paw in comparison with oil-treated group, from the third day after CCI (*p* < 0.0001).

Two-way ANOVA for repeated measures revealed a significant main effect for treatment (F1,18 = 60,032; *p* < 0.0001) and time (F4,72 = 9.154; *p* < 0.0001), but not for treatment × time interaction (F4,72 = 0.827; *p* = 0.5126). No significant difference in withdrawal of contralateral paws was revealed.

#### 2.2.2. Immunohistochemical Analysis

Immunofluorescence (IF) analysis, performed seven days after CCI (D7) in sections derived from sciatic nerve stained with CD11b antibody, shows a higher infiltration of macrophages in 17β-estradiol-treated females in comparison with control females (*p* = 0.0195) (Figure 4B).

#### 2.2.3. The Anti-Inflammatory Potential of 17β-Estradiol

The panel of 40 pro- and anti-inflammatory mediators was used to verify the effect of 17β-estradiol treatment on cytokines and chemokines in sciatic nerves lysates of female mice (Table 3). We observed a strong effect on the anti-inflammatory cytokine IL-13, which was up-regulated in CCI D7 females in comparison with naïve (Table 1) but this effect was stronger after 17β-estradiol treatment (Table 3). Furthermore, we observed that 17β-estradiol was able to revert and up-regulate several cytokines/chemokines important for macrophage activation and migration (MIG, eotaxin, eotaxin 2, KC, SDF-1, GM-CSF, TCA-3, MCSF), and for neuron and glia cells (fractalkine, SDF-1, exotoxin, BLC, MIG) (Table 3; Figure 4C). The upregulation of these chemokines is correlated with an activation of glia cells in the spinal cord, as confirmed by immunofluorescent analysis (previously shown). In addition, 17β-estradiol was able to increase the expression of LIX (also known as CXCL5), lymphotactin (also known as XCL1), GCSF, and to revert the expression of TNFα soluble receptors (Type I and II), which were down-regulated in CCI D7 females. On the other hand, 17β-estradiol treatment decreased the expression of BLC and I-TAC, a pro-inflammatory cytokine (Table 3).

The table shows only significant inflammatory mediators revealed in sciatic nerve samples of Naïve or CCI (D7) female mice treated with 17β-estradiol. Data are shown as FOLD CHANGE (CCI/NAÏVE). Any >1. 5-fold increase or <0. 65-fold decrease in signal intensity for a single analyte between samples may be considered a measurable and significant difference in expression. Values indicate the significant upregulation and downregulation versus naïve.

#### 2.2.4. Immunohistochemical Analysis: Role of 17β-Estradiol in the Expression/Activation of Spinal Glial Cells

Behavioral results have also been associated with IF analysis of specific markers at the spinal cord level. To analyze the role of 17β-estradiol in the expression (activation and morphology) of glial cells, we performed IF staining of astrocytes (GFAP) and microglia (CD11b) 7 days after CCI (D7). We observed a significant increase of reactive and hyperactive astrocytes in 17β-E female mice compared to OIL females (*p* = 0.0017; Figure 4D) and a significant increase in 17β-E female mice of (H/B) and (U/A) microglia in comparison to OIL group (*p* = 0.0006, *p* < 0.0001, respectively; Figure 4D). On the other hand, we observed a significant decrease of (R) resting, non-active microglia (*p* < 0.0001; Figure 4D) in 17β-E females compared to OIL female group. These data support the key neuromodulatory role of 17β-estradiol in NeP.

## 3. Discussion

In previous studies we demonstrated that male mice are highly different from females in terms of development of NeP, glia activation and functional recovery [16,17]. In the present paper, we provide evidence for the relevance of sex differences in the activation of immune system and onset of NeP, providing evidence for differential inflammatory response, location and distribution of immune cell population, adding important pieces of information on the role played by sex in chronic pain via the mediation of the immune system.

Gender-associated differences in chronic pain as well as in response to pharmacological therapy are widely reported in patients, with a higher susceptibility to nociceptive stimuli and more frequent use of analgesic/painkiller medications in women than in men [10,14,27,28,29,30,31]. Since in humans a number of factors, including sociocultural aspects, may contribute to elicit gender-related differences in pain processing, the use of animal models offers a productive approach for the understanding of some neurobiological mechanisms underlying the differences between women and men in chronic pain. Peripheral nerve injury induces structural and functional alterations of primary sensory neurons that are combined with a strong immune response of the somatosensory system. Injured neurons and glial cells release factors that activate resident immune cells and recruit more immune cells from circulation [32,33,34,35]. Signaling between the sensory and immune systems is bidirectional and this crosstalk is relevant in the design of new drugs for the treatment of chronic pain [36]. Following sciatic nerve injury, a series of events occurs at both peripheral and central levels that trigger inflammatory processes and, ultimately, neuropathic pain. Changes in nerves are related to an immediate immune response and recruitment of blood immune cells, with Schwann cells, resident and infiltrating macrophages releasing proinflammatory cytokines and chemokines [2,3,37,38].

Our data support sex-dependent differences in the activation of both innate and adaptive immune systems. When we analyzed the immune population by means of immunofluorescence and FACs in nerve lysates, we found sex-associated differences both in number and distribution of immune cells; in particular, we observed a higher infiltration of T cells in female nerves while macrophages were enhanced in males after injury. FACs also reveal that under basal condition sex-dependent differences are disclosed in nerve lysates, with regard to NK cell population that was less expressed in females. When plasma is analyzed by FACs, the same T cells and macrophages resulted less expressed after nerve injury, while female mice in baseline condition showed a higher resident T-lymphocytes population in comparison with males. Several reports have highlighted the pathophysiological association between peripheral immune cells infiltration, and in particular infiltration of CD3- or CD4-positive T lymphocytes, and perseverance of mechanical allodynia [39,40]. Here, the pro-nociceptive function of CD3-positive T lymphocytes infiltration in the sciatic nerve described in a gender-specific fashion the immune response to nerve injury in female mice, thus contributing to account for the greater hypersensitivity to allodynic stimulation at D3, D5 and D7 after CCI. Among the pro-inflammatory factors secreted upon nerve injury there are monocytes-derived macrophages that are identified as active contributors to pain exacerbation, particularly for NeP chronification [41,42]. Notably, hyperalgesia and pain sensitization can be suppressed by macrophages depletion [43,44], and we found major macrophage infiltration in the sciatic nerve of CCI male mice, that was higher than observed in CCI female mice. Thus, the induction of NeP drives both adaptive and maladaptive responses whose differential impact depends on the gender of the animal injured. Literature has focused its attention on the involvement of non-neuronal elements in pain transmission, showing that glia-derived signaling molecules can contribute to and modulate pain signaling in the spinal cord. In particular, microglia exert a regulatory action on NeP after nerve injury, and there is now extensive evidence that activation of CNS microglial cells is an essential part of pain hypersensitivity, as for instance for the contribution of BDNF expression in the neuron-microglia signaling system [45]. Astrocytes and microglia play a causative role in the development, spread and potentiation of neuropathic pain [46,47]; they respond to peripheral nerve injury with changes in morphology, proliferation, migration and expression of inflammatory regulators, activating reactive gliosis [48,49,50].

However, the role of microglial activation in the mediation of pain hyperalgesia is a sexually dimorphic effect [51], as further evidenced by the toll-like Receptor 4 (TLR4)-dependent regulation of microglia activation and NeP in male but not female mice [52]. Indeed, experimental ablation of microglia is able to reduce pain and reverse allodynia only in male mice [23]. The immunohistochemical analysis of glial markers in spinal cord disclosed several differences between male and female mice. While male mice showed an increase in the number of hyperactive astrocytes (GFAP positive cells) and hyperactive/bushy and/or unramified/ameboid microglia (CD11b positive cells), female mice did not show any early activation of astrocytes and microglia. From a previous study [16], we know that in females a delayed but long-lasting glia activation occurs, and that this late and persistent activation may lead to neurotoxicity and be detrimental for neural tissue regeneration [53]. If T cells infiltration within the site of peripheral injury is one key pathophysiological event underlying development of NeP in female sex, then this reactive immune response may possibly (at least in part) explain why females do not “require” microglia activation to elicit neuropathic hypersensitivity. The most elegant demonstration of this idea is probably the possibility to switch towards a “male way” of pain processing by female mice that underwent testosterone supplementation, which was shown to suppress T cells-mediated anti-allodynic effects induced by the stimulation of peroxisome proliferator activated receptors (PPARs) subtype γ (PPAR-γ), largely present in T cells [23]. Likewise, female mice lacking T cells will mimic the microglia-dependent “male way” of pain processing and then return back to microglial-independent pain processing after T cell production has been restored [23]. Notably, swim stress-induced analgesia (SSIA) is a gender-dependent phenomenon and ovariectomy can convert female’s analgesic response in male-like sensitivity to pharmacological disruption of SSIA [54]. This is an example of the powerful effects induced by estrogen depletion as well as of the estrogen-dependent mechanisms of pain processing. Hormone replacement therapy (HRT) is a clinical practice followed for several diseases such as in postmenopausal osteoporosis, prevention of bone density loss, osteoarthritis and pain perception [55,56,57]. It is worth mentioning that estrogen deficiency in postmenopausal co-morbid osteoporosis and rheumatoid arthritis dysregulate the immune system and T cells distribution, producing an increased expression of inflammatory cytokines such as IL-17 [58]. Moreover, the hypothesis that dysfunction of chloride channels and derangement of chloride conductance in the spinal cord and sensory afferent neurons may have a major role in the pathogenesis of NeP [59] appears corroborated by the anti-allodynic effects induced by the upregulation of voltage-gated chloride channel-3 (ClC-3) induced by 17β-estradiol delivery in ovariectomized rats [60].

Upon nerve damage and subsequent activation, several non-neuronal immune cells such as mast cells, Schwann cells and macrophages secrete a large number of soluble cytokines, damage-associated molecular patterns, as well as reactive oxygen species, nitric oxide and prostaglandins. In particular, a significant part of the orchestration of the inflammatory response, cytokines/chemokines secretion, tissue repairing and regulation of NeP are attributed to macrophage activation [42]. We previously demonstrated that after nerve injury several pro-inflammatory and anti-inflammatory cytokines/chemokines can be either up- or down-regulated in both serum and nerve lysates of male mice [25]. Here, while some changes occurred in the same fashion in male and female mice, as for the up-regulation of the pro-inflammatory IL-1β in sciatic nerves, other cytokines resulted up-regulated only in female mice or showed opposite levels of changes between the two sexes. The experiments revealed a tightly-regulated, sex-driven control of macrophage infiltration/activation, with much higher macrophage infiltration in injured peripheral nerves from male than female animals, whereas the macrophage marker F4/80 was concurrently found reduced in plasma of male mice. Such lower macrophage infiltration in female mice was accompanied by the upregulation of selected cytokines involved in the inflammatory signaling such as the IL12p40/70 that is involved in proliferation of cytotoxic T lymphocytes and NK cells, and regarded as cytokine enhancer of immune response [61]. We also detected the up-regulation of MIP1-α (aka CCL3), a pro-inflammatory cytokine that was found similarly enhanced in the blood of patients suffering from neuropathic pain [26]. Moreover, the selective up-regulation of I-TAC, MIG, SDF1 and TAC3 cytokines provides a possible mechanistic base for the higher allodynic response found in female mice, being that these cytokines are chemoattractants for different immune cells, and are involved in the development of tactile allodynia following peripheral injury through the enhancement of spinal nociceptive transmission and activation of glial cells [62]. Similarly, the analysis of the inflammatory array on blood lysates revealed only in neuropathic female mice the selective increase of T cell-secreted TCA-3 (thymus-derived chemotactic agent 3, also known as CCL1), which is also secreted by mast cells and endothelial cells, and plays an important role as a chemoattractant for neutrophils and monocytes and for the development of injury-induced tactile allodynia [62]. Moreover, female mice showed an increase not only of the potent T cell-produced inhibitor of inflammation IL-10, but also an increase of RANTES/CCL5 secretion, a chemokine with important chemoattractant features that is produced in response to inflammatory stimuli, as for instance described in patients with rheumatoid arthritis [63]. Considering that IL-10 synthesis has been shown to counteract microglia-derived RANTES/CCL5 [64], the up-regulation of IL-10 in female mice might be viewed as a defensive response against nerve lesion-induced RANTES/CCL5 production. Some pro-inflammatory mediators such as IL-1β and adipocytokine such as leptin are recognized as involved in the promotion of neural hyperexcitability, for instance by the binding to receptors placed at the spinal cord and dorsal root ganglion [65]. Indeed, recent evidence corroborates the view that adipocyte-derived leptin is involved in nociception, NeP development and allodynic response [66]. However, while changes in IL-1β regulation (namely, an up-regulation) were found similar in both sexes of nerve injured animals, variations in leptin regulation were conversely strictly gender-dependent. Of particular interest, we found an up-regulation of the adipocytokine leptin in lysates from sciatic nerves exclusively in neuropathic female mice. Sex hormones regulate leptin secretion, and there are several reports accounting for an inverse relationship between leptin and testosterone levels in the male gender [67,68] as well as association between body-pain and leptin dysregulation in postmenopausal woman [69]. The possible alteration of N-methyl-d-aspartate (NMDA) receptor-mediated signaling [70] or IL-1β regulation [71] are just two among the different mechanisms that have been suggested to account for leptin-mediated pain sensitization and NeP development. In addition to stimulating IL-1β release from microglia [71], leptin is also involved in the activation and proliferation of spinal microglia, thus contributing to its role in the development of NeP [72]. However, despite the involvement of leptin in microglia activation [73] and in modelling of astrocytes morphology [74], only male mice exhibited astrocytes (GFAP positive) and microglial cell (CD11b positive) activation. Thus, leptin was up-regulated only in female mice and not associated to astrocytes or microglia alterations. Our data corroborate the idea that the up-regulation of leptin signaling is a key element for both pathogenesis and maintenance of NeP, and that changes in leptin regulation could be a distinctive gender-dependent mechanism contributing to explain the higher allodynic response and the incomplete recovery from NeP observed in previous studies in female mice [16]. Better characterized is the role of leptin signaling in obesity-associated low-grade systemic inflammation [75]. Among the different factors and cell types, adipocytes also secrete specific cytokines such as MCP1 (aka CCL2) that is functionally associated to macrophage recruitment [76], and that we found specifically upregulated in neuropathic female mice. Since there is evidence that the increase of leptin synthesis facilitates MCP1 production [77], the up-regulation of leptin in neuropathic female mice may also contribute to explain the serum increase of MCP1 in the same animals. Along the same line of evidence, 17β-estradiol administration reverted in female mice the down-regulation of key cytokines involved in the migration and activation of macrophages (MIG, eotaxin, eotaxin 2, KC, SDF-1, GM-CSF) and glia cells (fractalkine, SDF-1, exotoxin, BLC, MIG). The down-regulation of these chemical signals observed in neuropathic female mice is associated with a decreased activation of macrophages and glial cells. Conversely, the up-regulation of these chemokines in 17β-estradiol-treated females is associated with macrophages activation in peripheral nerve and glia cells in the spinal cord, which may help to explain the marked decrease of allodynia in female mice that underwent 17β-estradiol administration.

The present study expands our view about the role of sexual dimorphism in pain processing. These data may contribute to improve the understanding of some neurobiological mechanisms underlying gender differences in NeP development. Sex-dependent immune and cytokines/chemokines changes are of key importance to identify neuropathy-associated inflammatory profiles and reliable biomarkers for more effective use of the current therapeutic agents and to design innovative and gender-oriented therapies.

## 4. Materials and Methods

### 4.1. Animals

CD1 male and female mice, about 3 months old from Charles River Labs (Como, Italy), were used. Animals were housed in standard transparent plastic cages, in groups of 4, lined with sawdust under a standard 12/12-h light/dark cycle (07:00AM/07:00PM), with food and water available ad libitum. Functional testing was performed blind as for the treatment group to which each subject belonged. After behavioral/functional testing, the estrous cycle was analyzed in females by the means of vaginal smears. Since we did not observe any difference in behavioral responses, we included all females in the same experimental group independently from the estrous cycle. Each experimental group consisted of 20 mice.

All procedures were in strict accordance with the current European and Italian National law (DLGs n.26, 4 March 2014, application of the European Communities Council Directive 2010/63/UE) on the use of animal for research and within the guidelines of the Committee for Research and Ethical Issues of IASP [78]. The experimental protocol was approved by the Italian Ministry of Health (Authorization n. 33/2014).

### 4.2. Surgery

Following the procedure originally proposed by Bennett and Xie [79], adapted to the mouse, the Chronic Constriction Injury (CCI) model was used as model of neuropathic pain. The CCI of the sciatic nerve was performed under anesthesia with chloral hydrate (500 mg/kg); the middle third of the right sciatic nerve was exposed through a 1.5 cm longitudinal skin incision. Three ligatures (5-0 chromic gut, Ethicon) were tied loosely around the sciatic nerve. The wound was then closed with 4–0 silk suture. In the following, the injured right hind paw will be named ipsilateral paw and the uninjured left hind paw will be named contralateral paw.

### 4.3. Drug

One hour after CCI, a group of female mice (*n* = 10) was treated with 17β-estradiol (ES) (Sigma-Aldrich), 50 μg/kg/day for seven days [17,80] and compared with a group of oil-injected mice (*n* = 10), as a control group.

### 4.4. Functional Testing

Mechanical allodynia. Neuropathy onset was assessed by measuring the sensitivity of both ipsilateral and contralateral hindpaws to normally non-noxious punctuate mechanical stimuli, at different time intervals from postoperative day 3 (D3) to D7. The nerve injury-induced mechanical allodynia was tested by using a Dynamic Plantar Aesthesiometer (Ugo Basile, Model 37400, Gemonio, Italy), an apparatus that generates a mechanical force linearly increasing with time. The force is applied to the plantar surface of the mice hindpaw, the nociceptive threshold is defined as the force (in grams) at which the mouse withdraws its paw. Each day of testing, the mechanical withdrawal response of ipsilateral and contralateral hindpaws was taken from three consecutive trials with at least 10 s between each trial. The withdrawal threshold was taken to be the mean of the three trials.

Thermal hyperalgesia. This parameter was assessed by measuring the withdrawal latency of both hindpaws from an infrared heat stimulus by using an automatic plantar test instrument based on the Hargreaves method (Plantar Test, Ugo Basile, Comerio, Italy). This apparatus basically consists of a movable infrared generator placed below a glass pane upon which animal is placed. A Perspex enclosure defines the space within which the animal is unrestrained. After an acclimation period, the infrared source placed under the glass floor was positioned directly beneath the hindpaw and trial begin. When the animal felt pain and withdrew its paw, the infrared source switched off and the reaction time counter stopped. The withdrawal latency to the nearest 0.1 s was automatically determined. To avoid damage of hindpaw skin tissue, a cut off time of 15 s was imposed. The test was performed at different time intervals from postoperative day 3 (D3) to D7 in both male and female mice.

### 4.5. Immunohistochemical Analysis

Sciatic nerves and spinal cords from mice belonging to each experimental group (*n* = 3/group) were harvested for immunofluorescence (IF) analysis. Animals were sacrificed with an overdose of chloral hydrate. Sciatic nerves were removed and kept in immersion for 48 h in 4% paraformaldehyde in phosphate buffer saline (PBS, pH 7.4) after cryoprotection with solution a of 30% (*w*/*v*) sucrose in PBS and maintained at −80 °C. Cryostat sections of sciatic nerve, 20 μm, were taken.

For sciatic nerve, IF analysis was made in no-CCI (naïve) and in CCI mice (D7: seven days after CCI). For IF staining, sections were first incubated overnight with: anti-CD3 (lymphocytes CD3 marker) antibody (rabbit polyclonal, 1:100, Santa Cruz); anti-CD11b (complement receptor 3/cluster of differentiation 11b, macrophages marker) antibody (rat anti-mouse, 1:100, Millipore); anti-mast cell chymase (Mast cell chymase marker) antibody (mouse monoclonal, 1:100, Santa Cruz) in Triton 0.3% (Sigma-Aldrich).

For the spinal cord, IF analysis was made in no-CCI (naïve) and in CCI-D7 mice. For IF staining, sections were first incubated overnight with: anti-GFAP (glial fibrillary acidic protein, astrocyte marker) antibody (mouse monoclonal, 1:100, Sigma-Aldrich) and anti-CD11b (complement receptor 3/cluster of differentiation 11 b, microglia marker) antibody (rat anti-mouse, 1:100, Millipore), in Triton 0.3%.

After triple washing in PBS, sections were incubated for 2 h at room temperature with fluorescein-conjugated goat anti-mouse (FITC, 1:100, Jackson ImmunoResearch), fluorescein-conjugated rat anti-mouse (FITC, 1:100, Jackson ImmunoResearch) or rhodamine conjugated goat anti-rabbit (TRITC, 1:100, Jackson ImmunoResearch) secondary antibodies in 0.3% Triton. After twice washing in PBS, sections were incubated for 10 min with bisBenzimide, DNA-fluorochrome (Hoechst, 1:1000, Sigma-Aldrich) in PBS.

Images of immunostained sections were acquired by laser scanning confocal microscopy using a TCS SP5 microscope (Leica Microsystem) connected to digital camera diagnostic instruments operated by I.A.S. software of Delta Systems Italia. Figures were assembled by means of Adobe Photoshop CS3 and Adobe Illustrator 10. Quantification was performed by means of the ImageJ software (version 1.41, National Institutes of Health, Bethesda, MD, USA). The number of CD3, CD11b and Mast cell positive cells was automatically counted in high (63×) magnification images of immunostained sections of sciatic nerves (two sections/animal) with the mark and count tool; the mean of three animals was calculated for each group of mice.

To evaluate astrocytes and microglia morphometric analysis in the spinal cord, high magnification images of the ipsilateral side of the dorsal horns for each animal were captured with a 63X objective at zoom factor 5 by using a constant set of acquisition parameters. Quantification was performed by means of the ImageJ software (version 1.41, National Institutes of Health, Bethesda, MD, USA).

### 4.6. Gliosis Evaluation and Morphometric Analysis

ImageJ quantification of GFAP and CD11b expression was (at least 2 slices for each animal) by converting pixels in brightness values on longitudinal section of spinal cord (magnification 5×) using RGB (red, green and blue) method, as described in Inman et al. (2005) [81]. The frame of rectangular areas of 100 mm, for a total length of 800 mm (from ipsilateral side of the dorsal horns direction), was singularly examined.

Morphometric analysis was performed on high-resolution imagine acquisition criteria: 63× objective, zoom 3×, 1024 × 1024 frame, 10 Hz. Each image was transformed in binary modality in order to obtain a digital image where the outline of cell silhouettes was identified and automatically measured for astrocytes, while microglia were individually counted and divided based on the different morphologies: Resting/Ramified (R), Hyperactive/Bushy (H/B), Unramified/Ameboid U/A).

### 4.7. Inflammatory Antibody Array

The differential level of expression of several cytokines/chemokines in serum and sciatic nerves lysates was analyzed using a mouse antibody array glass chip (Mouse Cytokine Antibody Array G series; RayBiotech) as previously described [82].

Briefly, Serum and nerve lysate samples were harvested from no-CCI (naïve), and CCI-D7 and CCI-D7 17-βEstradiol-treated female mice. Serum samples were diluted 5-fold with a blocking buffer. Lysis buffer (RayBiotech) containing proteinase inhibitor (Sigma-Aldrich) was added to homogenate sciatic nerves, protein concentration was determined, and 30 µg of each sample was added to the array. Incubation and washes were performed according to the manufacturer’s instructions. Glass chips were then incubated with biotin-conjugated primary antibody and fluorescent dye-conjugated streptavidin according to manufacturer’s instructions. Fluorescence detection was performed using an Agilent G2564B microarray scanner (Agilent Technologies) and analysis was performed using the array testing services from RayBiotech. (*n* = 3 each group × 3 replicates).

### 4.8. Sciatic Nerve Tissue Digestion for Flow Cytometry

As for previous experiments, sciatic nerves from naïve (no-CCI) and CCI-D7 male and female mice (*n* = 3/group for five times), were harvested for flow cytometry analysis. With the body placed on ice, sciatic nerve was collected into HBSS (Hank’s Balanced Salt Solution) with antibiotics (PEN-STREP and gentamicin) that remained on ice until processing. Briefly, sciatic nerve tissues were digested at 37 °C in RPMI (with 10% (*v*/*v*) fetal bovine serum (FBS) (Sigma-Aldrich), with 40 μL collagenase A (0.8 mg/mL) (Roche Diagnostics) and 100 μL deoxyribonuclease-1 (DNase-1, 400 units/μL) (Sigma-Aldrich). For optimal enzymatic digestion, tissue samples were incubated under slow continuous rotation using a rotator. Following digestion steps, cell suspensions were passed sequentially through 40 μm cell strainers (Corning™ sterile cell strainers, Fisher Scientific). Cells were collected from the interface and washed with RPMI at 400 g for 10 min at 20 °C. Cells were re-suspended in PBS and kept on ice before proceeding to viability dye staining.

### 4.9. Blood Preparation for Flow Cytometry

One mL peripheral blood, from deeply anesthetized naïve and CCI-D7 mice belonging to each experimental group (*n* = 3/group for five times), was collected (in BD vacutainer K2EDTA blood collection tube). Briefly, 300 μL of blood was diluted to 5 mL with lyse red blood cells (RBCs) and left for 10 min.

Cells were washed and diluted with 10 mL PBS (w/o Ca/Mg) in a 15 mL conical tube and centrifuged at 400× *g* for 10 min at 20 °C. Cells were re-suspended in PBS and kept on ice until proceeding to viability dye staining.

### 4.10. Flow Cytometry Analysis for Surface Immune Markers

Nerve and peripheral blood samples were stained with pre-defined optimal concentration of antibodies and Dead cells stain dyes (LIVE/DEAD Fixable Aqua Dead Cell Stain Kit, Life Technologies). The following anti mouse antibodies were used: CD45 APC-eFluor-780 conjugated (eBioscience) to select hematopoietic lineage cells; CD335 PE-eFluor 610 conjugated (eBioscience) characteristic of Natural Killer cells; CD3 PE conjugated (Miltenyi) to identified T lymphocytes; CD45R VioBlue conjugated (Miltenyi) to identified B lymphocytes; F4/80 PE Vio770 conjugated and MHC-II Fitc conjugated (Miltenyi) to select monocyte population. After 20 min of staining the cells were washed twice with Phosphate Buffered Saline (PBS) and then analyzed to flow cytometer equipped with 3 lasers (Cytoflex -Beckman Coulter). Cytoflorimetric analysis was performed with FlowJo software (FlowJo, LLC Company Ashland, OR, USA).

### 4.11. Data Analysis

All values are expressed as mean ± SEM. One-way ANOVAs or two-way ANOVAs for repeated measures were used to analyze immunohistochemical data or the effects of sex-related behavioral differences, respectively. When necessary, post hoc comparisons were carried out using Tukey–Kramer test. Differences were considered significant at *p* < 0.05.

## Figures and Tables

**Table 1:** Cytokines and chemokines expression on sciatic nerves tissue lysates of CCI female mice.

Up-Regulated Inflammatory Mediators	Fold Change
CD30 L	2.662401
Fas Ligand	5.486272
GCSF	2.509602
IL-1 beta	3.844157
IL-10	1.850482
IL-12p40/70	4.466972
IL-13	3.448072
I-TAC	2.320823
KC	2.860148
Leptin	2.360061
MIP-1 alpha	3.051168
**Down-Regulated Inflammatory Mediators**	**Fold Change**
BLC	0.210316
Eotaxin-2	0.207665
Fractalkine	0.444928
GM-CSF	0.620337
MIG	0.139313
SDF-1	0.635213
sTNF R I	0.275478
sTNF R II	0.036147

**Table 2:** Cytokines and chemokines expression in serum samples of CCI female mice.

Up-Regulated Inflammatory Mediators	Fold Change
IFNgamma	1.411082
IL-1 alpha	4.337264
Il-6	1.558615
IL-10	2.03
IL-12p40/70	2.577468
I-TAC	3.445911
KC	1.50214
MCP-1	1.835833
MIG	2.505671
MIP-1 alpha	1.840265
RANTES	2.197797
SDF-1	2,423905
TCA-3	1.920029
TIMP-2	2.517015
**Down-Regulated Inflammatory Mediators**	**Fold Change**
Fas Ligand	0.372778
Leptin	0.416235

**Table 3:** Cytokines and chemokines expression on sciatic nerves tissue lysates of CCI 17β-E female mice.

Up-Regulated Inflammatory Mediators	Fold Change
CD30 L	5.429201
Eotaxin	5.758793
Eotaxin-2	2.123679
GCSF	10.30591
IL-1 beta	4.767676
IL-12p40/70	4.572974
IL-12p70	4.63888
IL-13	13.68566
KC	3.676447
Leptin	2.198192
LIX	3.303392
Lymphotactin	1.599921
MCSF	6.939658
MIG	1.90799
MIP-1 alpha	4.137147
MIP-1 gamma	2.987606
RANTES	6.72374
SDF-1	2.768936
TCA-3	2.040783
TIMP-1	17.92104
TIMP-2	1.965147
sTNF R I	8.026424
sTNF R II	1.981349
**Down-Regulated Inflammatory Mediators**	**Fold Change**
BLC	0.654138
I-TAC	0.626375

## Data Availability

The data presented in this study are available on request from the corresponding author.
